# Structural analysis of the interaction between human cytokine BMP-2 and the antagonist Noggin reveals molecular details of cell chondrogenesis inhibition

**DOI:** 10.1016/j.jbc.2023.102892

**Published:** 2023-01-13

**Authors:** Charly Robert, Frédéric Kerff, Fabrice Bouillenne, Maxime Gavage, Marylène Vandevenne, Patrice Filée, André Matagne

**Affiliations:** 1 Laboratory of Enzymology and Protein Folding, University of Liège, Liège, Belgium; 2 Centre for Protein Engineering, InBioS Research Unit, University of Liège, Liège, Belgium; 3 Biological Macromolecule Crystallography, University of Liège, Liège, Belgium; 4 Analytical Laboratory, CER Groupe, rue du Point du Jour, Marloie, Belgium; 5 Laboratory of immuno-biology, CER Groupe, Novalis Science Park, Aye, Belgium

**Keywords:** bone morphogenetic protein (BMP), cell differentiation, chondrogenesis, Noggin, protein folding, protein-protein interaction, protein structure, recombinant protein expression, site-directed mutagenesis, X-ray crystallography, ALP, alkaline phosphatase, BLI, bio-layer interferometry, BMP, bone morphogenetic protein, CV-2, Crossveinless 2, IBs, inclusion bodies, RT-qPCR, reverse transcription-quantitative PCR, SEC-MALS, size-exclusion chromatography with multi-angle light scattering, TGF-β, transforming growth factor-β, UHPLC-HRMS, ultra-high-performance liquid chromatography coupled to high resolution mass spectrometry

## Abstract

Bone morphogenetic proteins (BMPs) are secreted cytokines belonging to the transforming growth factor-β superfamily. New therapeutic approaches based on BMP activity, particularly for cartilage and bone repair, have sparked considerable interest; however, a lack of understanding of their interaction pathways and the side effects associated with their use as biopharmaceuticals have dampened initial enthusiasm. Here, we used BMP-2 as a model system to gain further insight into both the relationship between structure and function in BMPs and the principles that govern affinity for their cognate antagonist Noggin. We produced BMP-2 and Noggin as inclusion bodies in *Escherichia coli* and developed simple and efficient protocols for preparing pure and homogeneous (in terms of size distribution) solutions of the native dimeric forms of the two proteins. The identity and integrity of the proteins were confirmed using mass spectrometry. Additionally, several *in vitro* cell-based assays, including enzymatic measurements, RT-qPCR, and matrix staining, demonstrated their biological activity during cell chondrogenic and hypertrophic differentiation. Furthermore, we characterized the simple 1:1 noncovalent interaction between the two ligands (*K*_D_*ca.* 0.4 nM) using bio-layer interferometry and solved the crystal structure of the complex using X-ray diffraction methods. We identified the residues and binding forces involved in the interaction between the two proteins. Finally, results obtained with the BMP-2 N102D mutant suggest that Noggin is remarkably flexible and able to accommodate major structural changes at the BMP-2 level. Altogether, our findings provide insights into BMP-2 activity and reveal the molecular details of its interaction with Noggin.

Bone morphogenetic proteins (BMPs) are multifunctional cytokines belonging to the transforming growth factor-β (TGF-β) superfamily. Although they were discovered as osteoinductive compounds (1), they play critical roles in the development and maintenance of various tissues in vertebrates and invertebrates, by regulation of cell proliferation, differentiation, and death (2, 3, 4). How BMP signaling regulates the formation and maintenance of various organs *in vivo*, in a highly context-dependent manner (5), is not completely understood. This is largely due to their biological activities being tightly regulated by an intricate combination of factors, *e.g.*, antagonists and potentiators, during various physiological and also pathological processes. Since BMPs take part in many cellular mechanisms, the deregulation of their signaling pathways can lead to various dysfunctions, affecting, for example, the cardiovascular (6), central nervous (7), and osteoarticular systems (8). To date, nineteen members of the BMP family have been identified, which show homologous 3D structures and significant sequence identity (9). Thus, BMPs generally are homodimeric molecules (10, 11), with an interchain disulphide bond holding the subunits together. Each subunit is made up of eight β-strands, disposed in two pairs of antiparallel β-sheets, and two α-helices (12), and it contains three intrachain disulphide bonds that form the distinct cystine-knot motif of the TGF-β superfamily.

BMP signaling occurs through binding to and subsequent oligomerization of two type I and two type II serine-threonine kinase receptors in the cell membrane, to form a heterohexameric complex (10, 13). This assembly ensures signal transduction through a phosphorylation cascade (14) involving Smad proteins, which form a complex that accumulates in the nucleus and mediate gene transcription (15). In addition, BMPs also activate Smad-independent signaling, such as mitogen-activated protein kinase pathways (16). Finally, both Smad-dependent and Smad-independent signaling pathways are upregulated and downregulated by various cellular effectors, such as coreceptors and secreted antagonists. Thus, the former are membrane-bound proteins, known as auxiliary accessory receptors or coreceptors (4), which fine-tune signaling by promoting or inhibiting the interaction of BMP ligands to their authentic receptors (17, 18, 19, 20), whereas the latter (*e.g.*, Noggin and Gremlin) bind to BMP receptor recognition sites (3, 21, 22, 23), hence physically preventing binding.

Within the BMP family, BMP-2 is one of the most studied members. It has been reported to play an important role in cartilage and bone formation (24, 25). Thus, in a process known as chondrogenesis, BMP-2 induces the differentiation of mesenchymal stem cells into mature chondrocytes, which are responsible for cartilage synthesis and regeneration. Moreover, BMP-2 also promotes the differentiation of mature chondrocytes into hypertrophic chondrocytes, which are involved in cartilage degradation and mineralization towards bone formation. In this context, the antagonist Noggin has been shown to inhibit BMP signaling and hence differentiation of stem cells into mature chondrocytes (26, 27). Chondrogenesis occurs during both articular cartilage formation and endochondral ossification, *i.e.*, a multistage differentiation process by which long bones are generated. Contrary to endochondral ossification, mesenchymal stem cells of a healthy articular cartilage differentiate into mature articular chondrocytes but do not undergo hypertrophic differentiation. Thus, articular chondrocytes remain quiescent and display only moderate metabolic activity to maintain the cartilage matrix. Malfunction in chondrogenesis can, however, lead to various disorders, such as articular cartilage degradation in osteoarthritis where chondrocytes undergo unexpected hypertrophy differentiation leading to cartilage degradation, apoptosis, and mineralization (28, 29).

Remarkably, BMPs have shown clinical potentials in cartilage and bone repair (30, 31, 32), and two of them (*i.e.* BMP-2 and BMP-7) have been approved by the U.S. Food and Drug Administration for therapeutic application in this context. Thus, BMP-2 in particular has been used for diverse therapeutic purposes, and both success and pitfalls have been reported. As for the latter, many side effects (33, 34)have been observed, notably bone ectopic formation, inflammatory complications, and tumor development. These observations prompt to both a better understanding of the structure and function relationships in BMPs and a comprehensive description of their interaction network.

This study focuses on the interaction between BMP-2 and the antagonist Noggin, in the context of chondrogenesis, with the aim of deepening our understanding of the interaction network of BMPs. The structural and functional properties of both proteins were analyzed using different and complementary techniques, allowing further functional analysis of the interaction of BMP-2 with the antagonist Noggin. In particular, study of the BMP-2 N102D mutant suggests that the antagonist molecule has a remarkable structural flexibility, which allows to accommodate structural changes of the cytokine.

## Results

### Production of recombinant BMP

**Figure 1:**
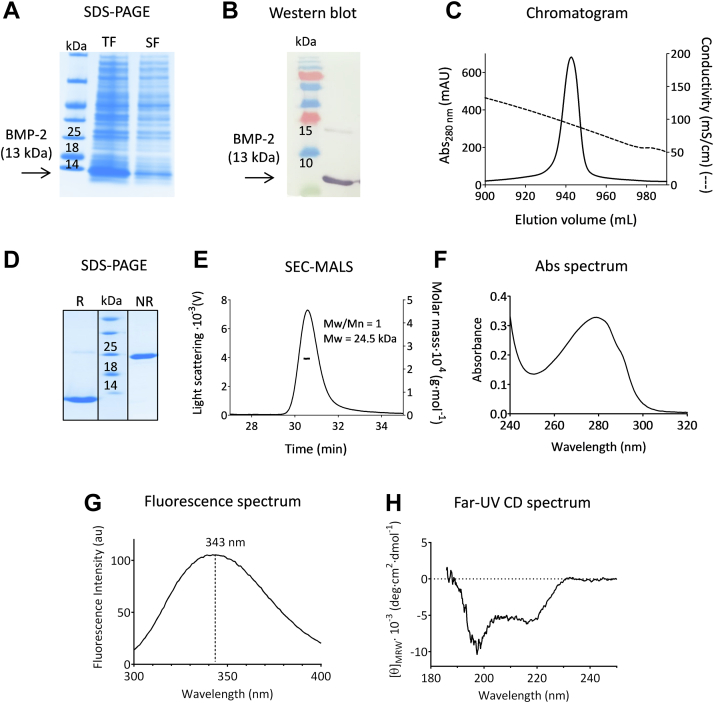
**Production of recombinant BMP-2.** Proteins were analyzed following separation on 4 to 20% SDS-PAGE combined with Coomassie blue staining (*A* and *D*) or with western blot analysis using anti-BMP-2 antibody (*B*). Lanes 1 to 3 in (*A*) correspond to the molecular weight marker and the total (TF) and soluble (SF) fractions from induced cells, respectively. Lanes 1 and 2 in (*B*) correspond to the molecular weight marker and the total fraction, respectively. Lane 2 in (*D*) corresponds to the molecular weight marker, whereas lanes 1 and 3 are for the purified, refolded protein, under reducing (R) and nonreducing (NR) conditions, respectively. BMP-2 was purified on a Source 15 ISO (isopropyl) column and the chromatogram is shown in (*C*), with mAU standing for milli absorbance units. The purified BMP-2 sample in 50 mM Tris–HCl, pH 7, 5 mM EDTA, 0.5 M arginine was analyzed by (*E*) SEC-MALS (protein concentration of ca. 1 mg mL^−1^), (*F*) UV absorbance (protein concentration of ca. 0.2), and (*G*) intrinsic fluorescence emission (protein concentration of ca. 0.1 mg mL^−1^). Far-UV CD measurements (*H*) were performed in 20 mM sodium phosphate buffer, pH 7, using a protein concentration of ca. 0.1 mg mL^−1^. In (*E*), Mw and Mw/Mn stand for molecular mass and polydispersity index, respectively. In (*G*), the wavelength corresponding to the maximum fluorescence emission intensity is indicated. BMP, bone morphogenetic protein; SEC-MALS, size-exclusion chromatography with multi-angle light scattering.

Expression of BMP-2 in *Escherichia coli* resulted in the aggregation of the protein molecules into inclusion bodies (IBs). Thus, Figure 1, *A* and *B* shows the occurrence, in the insoluble fraction mainly, of a protein with the expected apparent molecular mass (*ca*. 13 kDa) and specifically recognized by a mouse anti-BMP2 mAb. Following extraction and solubilization of BMP-2 IBs, refolding was achieved in one single dilution step and purification was performed on a hydrophobic interaction Source 15 ISO column. The elution profile (Fig. 1*C*) shows that BMP-2 elutes in a single sharp and symmetrical peak, suggesting the occurrence of one predominant isoform.

The quality of the final BMP-2 sample in 50 mM Tris–HCl, pH 7, 5 mM EDTA, 0.5 M arginine was assessed according to the best practice recommendation established by the ARBRE-MOBIEU and P4EU networks (35, 36) (see https://arbre-mobieu.eu/guidelines-on-protein-quality-control/). Thus, the purity was checked by SDS-PAGE (Fig. 1*D*) in both reducing and nonreducing conditions and was found to be above 98%. In both cases, a single band was observed, which corresponds to an apparent molecular mass of 13 and 26 kDa, respectively, and suggests that BMP-2 is purified as a disulphide-bonded dimer. Size-exclusion chromatography with multi-angle light scattering (SEC-MALS) (Fig. 1*E*) showed elution of BMP-2 as a homogeneous monodisperse species (*i.e.* no aggregation), with an apparent molecular mass of 24.5 kDa. Identity and integrity were confirmed by intact protein mass determination (UHPLC-HRMS), which revealed one major species corresponding to a molecular mass of 26,056.9 Da. This value is identical within the error limit to the value expected from the amino acid sequence of the protein (calculated *M*_r_ 26057.7). The absence of nucleic acid contamination was checked by UV absorbance measurements (Fig. 1*F*), and the occurrence of a natively folded protein was demonstrated by both circular dichroism and biological activity measurements (see below). Finally, BMP-2 was validated as endotoxin free by Lonza Testing Services, allowing its use in *in vitro* cell-based assays. Note that two different BMP-2 preparations were used in this study, and both minimal and extended quality control tests (36) were used to establish batch-to-batch consistency (see also discussion below).

Overall, the production (*i.e.* expression, refolding, and purification) protocol used in this study yields *ca*. 100 mg of pure, homogeneous, native, and fully functional BMP-2 per liter of cell culture.

### Production of recombinant Noggin

**Figure 2:**
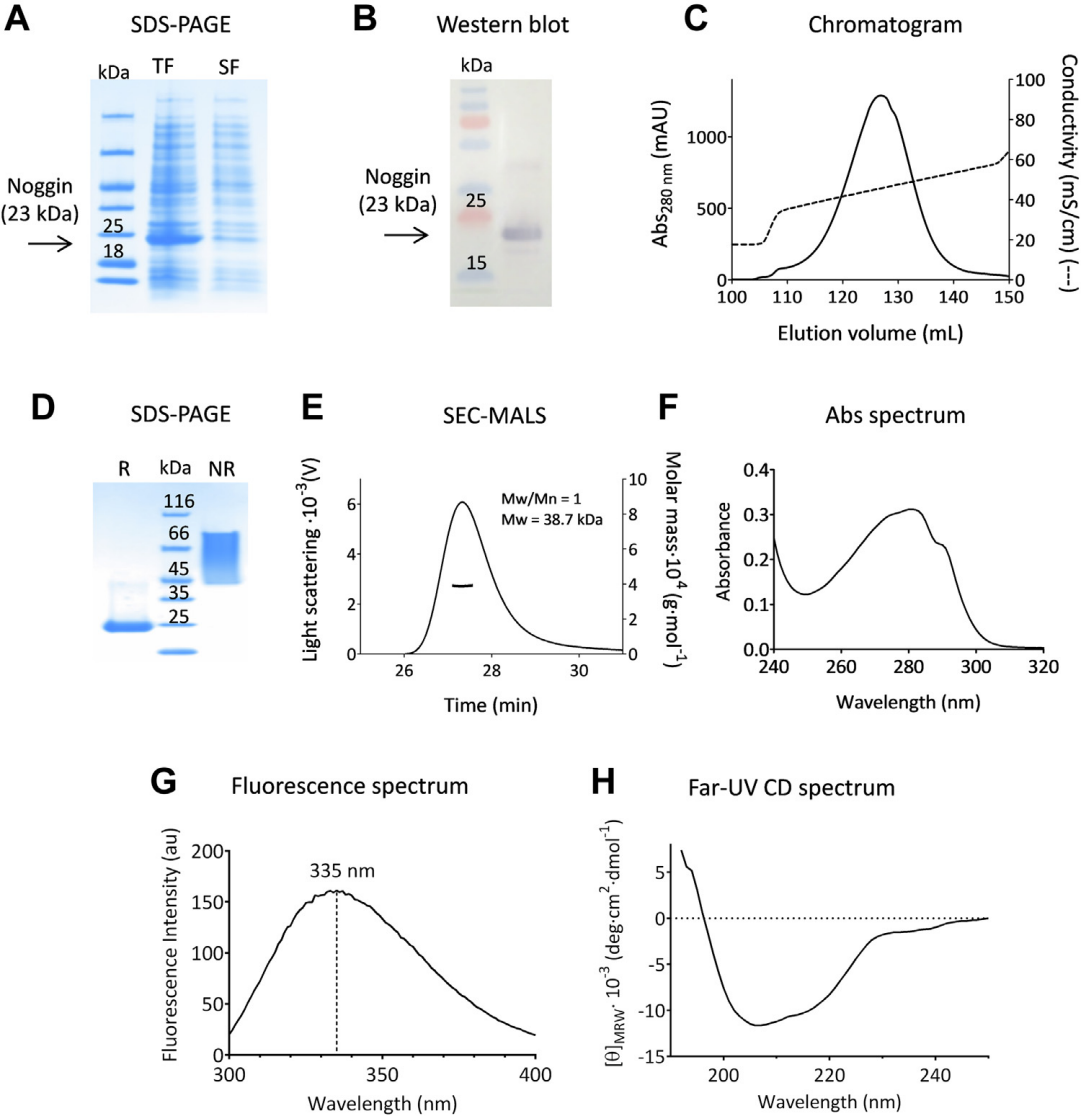
**Production of recombinant Noggin.** Proteins were analyzed following separation on 4 to 20% SDS-PAGE combined with Coomassie blue staining (*A* and *D*) or with western blot analysis using anti-Noggin antibody (*B*). Lanes 1 to 3 in (*A*) correspond to the molecular weight marker and the total (TF) and soluble (SF) fractions from induced cells, respectively. Lanes 1 and 2 in (*B*) correspond to the molecular weight marker and the total fraction, respectively. Lane 2 in (*D*) corresponds to the molecular weight marker, whereas lanes 1 and 3 are for the purified, refolded protein, under reducing (R) and nonreducing (NR) conditions, respectively. Noggin was purified on a HiTrap SP Sepharose FF column and the chromatogram is shown in (*C*), with mAU standing for milli absorbance units. The purified Noggin sample in 50 mM sodium citrate, pH 6.8, 150 mM NaCl, 1 mM magnesium acetate, 20% glycerol, 0.02% CHAPS was analyzed by (*E*) SEC-MALS (protein concentration of ca. 1 mg mL^−1^), (*F*) UV absorbance (protein concentration of ca. 1 mg mL^−1^), and (*G*) intrinsic fluorescence (protein concentration of ca. 0.2 mg mL^−1^). Far-UV SRCD measurements (*H*) were performed on the DISCO beamline of synchrotron SOLEIL, using a protein concentration of ca. 0.9 mg mL^−1^. In (*E*), Mw and Mw/Mn stand for molecular mass and polydispersity index, respectively. In (*G*), the wavelengths corresponding to maximum fluorescence emission intensities are indicated. SEC-MALS, size-exclusion chromatography with multi-angle light scattering.

Noggin was overproduced in *E. coli* as IBs (Fig. 2, *A* and *B*). Following protein expression, IBs were extracted, solubilized, refolded, and finally purified using an ion exchange HiTrap SP Sepharose FF column. The chromatogram shown in Figure 2*C* suggests the presence of one predominant isoform of Noggin as the protein elutes in a single sharp and symmetrical peak.

Quality control of Noggin in 50 mM sodium citrate, pH 6.8, 150 mM NaCl, 1 mM magnesium acetate, 20% glycerol, 0.02% CHAPS was performed as described for BMP-2, to verify purity, homogeneity, identity and integrity. Thus, SDS-PAGE (Fig. 2*D*) in reducing conditions showed a single band at an apparent molecular mass of 23 kDa, as expected for a Noggin monomer, and showed purity above 98 %. In contrast, under nonreducing conditions, Noggin migrates as a spread band corresponding to a molecular mass range of 45 (compatible with a disulphide-linked dimeric protein) to 80 kDa. This apparently artefactual behavior (see below) was already described for native Noggin (37) and remains unexplained. SEC-MALS (Fig. 2*E*) shows that Noggin was eluted as a homogeneous species (*i.e.* no aggregation), with an apparent molecular mass of 38.7 kDa. Intact protein mass analysis yielded three major peaks at 46,338.3 Da, 46,354.5 Da, and 46,370.6 Da. The first lower mass species was attributed to the Noggin dimeric form (expected average mass of 46,339.0 Da). The two other peaks were attributed (and this was confirmed by peptide mapping analysis; data not shown) to methionine oxidation, mainly on the N-terminal methionine. Thus, the peaks at 46,354.5 Da and 46,370.6 Da correspond to Noggin (dimeric), with a single (expected average mass of 46,355.0 Da) and a double (expected average mass of 46,371.0 Da) oxidation, respectively. Finally, UV absorbance spectroscopy (Fig. 2*F*) demonstrated the lack of significant contamination by nucleic acids and confirmed the absence of large protein aggregated species. Noggin was also validated as endotoxin free (Lonza Testing Services), allowing its use in *in vitro* cell-based assays.

The production (*i.e.* expression, refolding, and purification) protocol used in this study yields *ca*. 15 mg of pure, homogeneous, native, and fully functional Noggin per liter of cell culture.

### Fluorescence and CD spectroscopy

Both BMP-2 and Noggin display broad fluorescence emission spectra (Figs. 1*G* and 2*G*), with maxima at 343 and 335 nm, respectively. These values, which are significantly lower (*i.e.* blue shifted) than the emission maximum (near 355 nm) of solvent-exposed tryptophan, indicate reduced solvent exposure of the indole side-chains and hence suggest the existence of stable tertiary contacts within the protein molecules. Moreover, for BMP-2, a maximum at 343 nm is a characteristic feature of the native protein spectrum (38).

Analysis of the far-UV CD spectrum of each protein revealed a well-defined secondary structure. Thus, the spectrum of BMP-2 (Fig. 1*H*), with an ellipticity signal around zero at 230 nm and two ellipticity minima at 217 and 197 nm, shows the distinct signature of the native protein (21, 38). Calculation of the secondary structure content yielded (4 ± 1.5) % helix, (37 ± 5) % strand, (22 ± 3) % turn, and (37 ± 3) % unordered. As for Noggin, data shown in Figure 2*H* are also consistent with previous results (37) and allowed calculation of (6 ± 3) % helix, (33 ± 2) % strand, (25 ± 1.5) % turn, and (34 ± 2) % unordered. Together, these results suggest that the two refolded proteins display well-organized secondary and tertiary structures. With both proteins, however, decomposition of the far-UV CD spectrum gave very low α-helix content. These results are not consistent with the signal values measured at 222 nm, which allow the calculation (39) of *ca.* 18 and 23% α-helices for BMP2 and Noggin, respectively. For further discussion on the protein secondary structures, see below.

### Bio-layer interferometry

**Figure 3:**
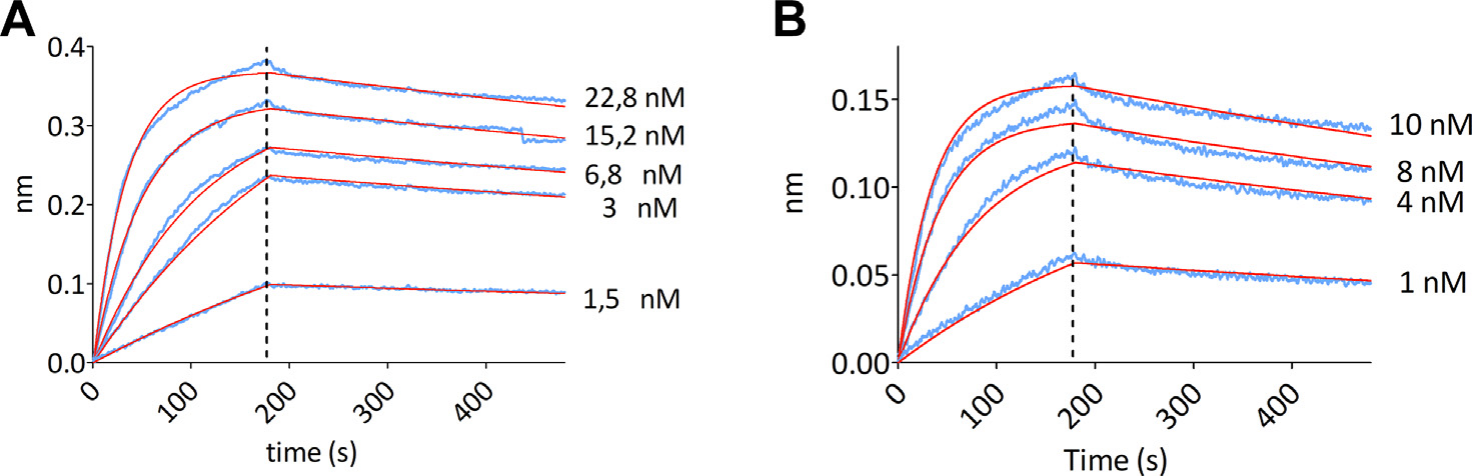
**Characterization of the complex between BMP-2 and Noggin.** Sensorgrams (in *blue*) obtained from bio-layer interferometry analysis of the binding of (*A*) WT and (*B*) N102D BMP-2 to Noggin show the association and dissociation kinetics, at 30 °C, pH 6.8, of BMP-2 solutions at different concentrations (as indicated) to biotinylated Noggin*;* the curves (in *red*) are the result of fitting a simple 1:1 binding model to the data (all R^2^ values > 0.99). Kinetic association and dissociation constants (*i.e.*, k_*a*_ and k_*d*_*, respectively*) as well as the resulting equilibrium dissociation constant (*K*_D_) values were obtained based on three repetitions of such an experiment (see text for details). The *vertical dotted line* indicates the switch from the association to the dissociation regimes. BMP, bone morphogenetic protein.

The kinetic rate constants for the association (*k*_a_) and dissociation (*k*_d_) of the BMP-2:Noggin complex were determined using a bio-layer interferometry (BLI) detection system (Octet HTX). Biotinylated Noggin was immobilized on streptavidin-coated biosensors and the kinetic parameters were obtained using five different BMP-2 concentrations. Data (Fig. 3*A*) show that BMP-2 interacts with Noggin according to a 1:1 reaction stoichiometry, with *k*_a_ and *k*_d_ values of (1.2 ± 0.1)·10^6^ M^−1^ s^−1^ and (4.2 ± 0.14)·10^−4^ s^−1^, respectively, yielding a *K*_D_ value of 0.36 ± 0.07 nM, in good agreement with that (0.6 nM) obtained using surface plasmon resonance (40).

### X-ray crystal structure of the antagonist Noggin bound to BMP-2

**Figure 4:**
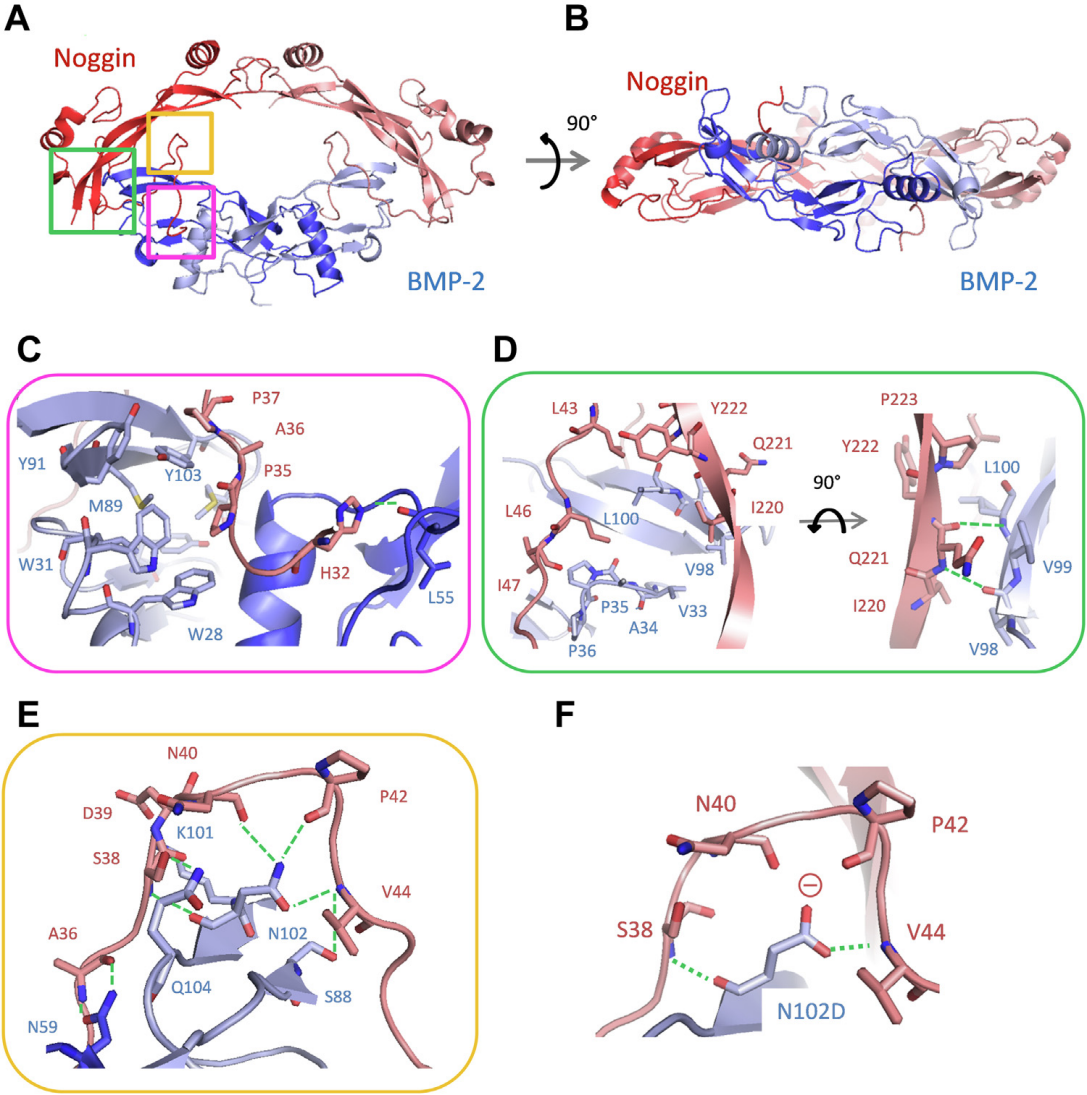
**The X-ray crystal structure of BMP-2 complexed with Noggin (Protein Data Bank code 7AGO) reveals three main contact regions.***A*, ribbon representation of the complex, where a BMP-2 dimeric molecule is shown (in *dark* and *light blue* to distinguish between the two monomers) bound to a Noggin dimeric molecule (one monomer in *red*, the other in *pink*), with the three main contact regions framed. *B*, same as (*A*) with a 90° rotation around the horizontal axis. *C*–*E*, enlarged view of the three contact regions at the binding interface, corresponding to the areas in the three boxes in (*A*). The side chains of residues constituting the binding interface are shown and electrostatic bonds are represented as *green dashed lines*. *F*, molecular model of the BMP-2 N102D mutant, which highlights the loss of two hydrogen bonds (with residue N40 and P42) and the predicted electrostatic repulsions around the carboxylate group of D102 (shown by Ɵ). Figures were generated using the open-source molecular graphics system PyMOL (The PyMOL Molecular Graphics System, Version 4.5.0., Schrödinger, LLC). BMP, bone morphogenetic protein.

Analysis of the complex (obtained as described in the Experimental procedure section) by SEC-MALS (Fig. S1) revealed a single, well defined, symmetric peak, corresponding to a homogeneous species with an apparent molecular mass of 64 kDa, in reasonable agreement with the expected value (*ca.* 72 kDa). Crystals of the complex belong to the P6_1_22 space group and allowed the structure (Fig. 4) to be solved at a resolution of 3.1 Å by using X-ray diffraction. The model is characterized by R_work_ and R_free_ values of 0.232 and 0.280, respectively (see Table S1 for additional crystallographic data and refinement statistics). The asymmetric unit contained one BMP-2 subunit (*i.e.* monomer) in interaction with one Noggin subunit, and the complete BMP-2:Noggin biological assembly (*i.e.* dimer:dimer) was generated from the two-fold symmetry axis.

Both BMP-2 and Noggin are covalently linked homodimers (10, 22) and display the characteristic cystine knot motif, conserved in all members of the TGF-β superfamily (41, 42). Each BMP-2 monomer contains two α-helices and five β-strands, organized in two pairs of antiparallel β-sheets, and are stabilized by three intrachain disulphide bridges (C14-C79, C43-C111, and C47-C113), which form the knot. In addition, the thiol group of a seventh Cys (C78) allows the formation of an interchain disulphide bridge, which holds the two monomers together and hence contributes to the stability of the protein quaternary structure. Noggin has a similar overall topology, with two identical monomers bridged by an interchain disulphide bond (C155). Each monomer is stabilized by four intrachain disulphide bridges, three of them (C155-C192, C178-C228, and C184-C230) forming a cystine knot. Further analysis of the X-ray crystal structure of the complex indicates that BMP-2 structural content is about 15% helix and 33% strand, while Noggin consists of *ca.* 18% helix and 32% strand.

Using the PDBePISA server (http://www.ebi.ac.uk/pdbe/pisa/), main crystal contacts were identified at the BMP-2 dimerization interface (2400 Å^2^), the Noggin dimerization interface (900 Å^2^), and the BMP-2:Noggin binding interface (2600 Å^2^) (Fig. 4). Thus, Noggin covers around one-third of BMP-2 residues (*i.e.* 74 out of 228) and interacts through four large hydrophobic patches, 22 hydrogen bonds, and 2 ionic bonds in total. Finally, the overall structure reveals that Noggin masks both type 1 and 2 receptor-binding sites. Thus, the core of Noggin interacts with the type 2 receptor-binding site, while its N-terminal segment inserts into the type 1 receptor-binding site. This interaction shows that Noggin, similarly to other known antagonists (21, 22, 43, 44, 45), precludes BMP-2 from binding to type 1 and 2 receptors and hence prevents any signaling cascade.

### BMP-2:Noggin–binding site

The structure of the antagonist Noggin bound to BMP-2 reveals that the binding interface can be divided into three contact regions (Fig. 4). Thus, a short hydrophobic segment (P35, A36, P37, Fig. 4*C*) of Noggin occupies the type 1 receptor-binding site of BMP-2, which consists of a large concave hydrophobic pocket (46). The latter is composed of residues W28, W31, I32, Y38, I62, M89, Y91, Y103, M106 and the contact surface is mostly hydrophobic. However, the interaction between the two proteins is further stabilized by a hydrogen bond between the Noggin H32 imidazole side chain and the BMP-2 L55 backbone carbonyl (Fig. 4*C*).

At the level of the type 2 receptor-binding interface (Fig. 4*D*), a concave hydrophobic structural element of Noggin, made up of six hydrophobic side chains (L43, L46, I47, I220, Y222, P223), interacts with two short convex hydrophobic segments of BMP-2 (*i.e.* V33 to P36 and V98 to L100). In addition, the backbone amine and carbonyl groups of Noggin Q221 make two hydrogen bonds with the backbone amides of BMP-2 V98 and L100 (Fig. 4*D*). This contact region covers the type 2 receptor-binding site of BMP-2.

Finally, the third contact region (Fig. 4*E*) involves a segment of BMP-2, which is located between the type 1 and type 2 receptor-binding sites. At this level of the contact interface between the two proteins, Noggin and BMP-2 interact through an extensive network of electrostatic interactions, involving a total of eight hydrogen and one ionic bonds. Thus, the amide side chain of BMP-2 N59 forms two hydrogen bonds with the backbone carbonyl and amide groups of Noggin A36; the hydroxyl group of BMP-2 S88 is hydrogen bonded to the backbone carbonyl of Noggin V44; BMP-2 N102 makes a total of four hydrogen bonds with Noggin residues S38, N40, P42, and V44; the amide side chain of BMP-2 Q104 forms one hydrogen bond with the hydroxyl group of Noggin S38; and, finally, the ε-amine of BMP-2 K101 makes a salt bridge with the carboxylate group of Noggin D39 (Fig. 4*E*).

### Biological characterization

The biological activity of both BMP-2 and Noggin was tested using the chondrogenic mouse ATDC5 cell line, which has been described as a predifferentiated cell line (47) that differentiates through a sequential process analogous to chondrocytes. Thus, ADTC5 cells differentiate first into mature chondrocytes, which are responsible for articular cartilage synthesis, and then into hypertrophic chondrocytes, which promote mineralization (47). This sequential process was monitored using three complementary, *in vitro*, cell-based assays, which specifically identify mature and hypertrophic phenotypes.

**Figure 5:**
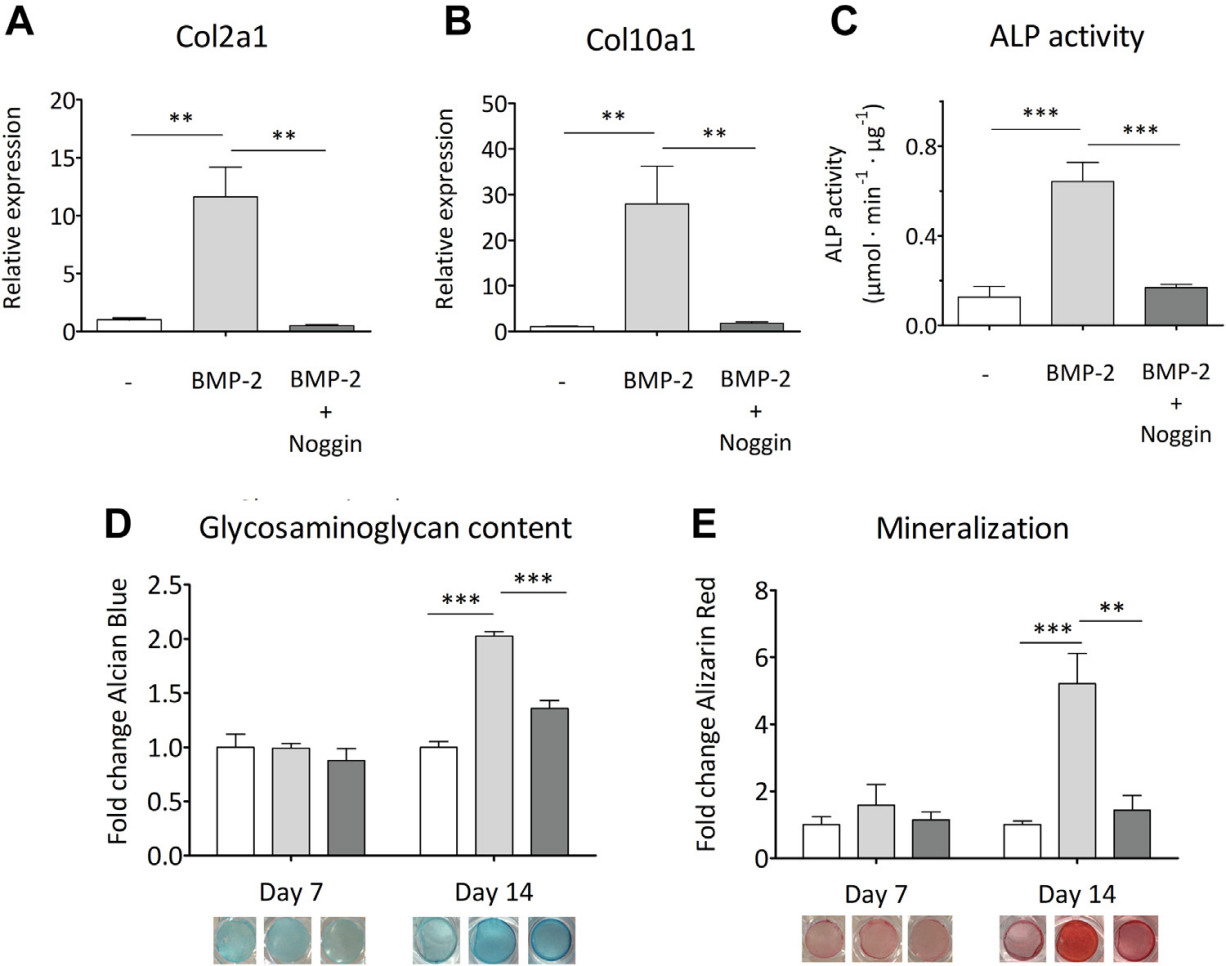
**Effects of BMP-2 and Noggin on the chondrogenic differentiation of ATDC5 cells.** Col2a1 (*A*) and Col10a1 (*B*) gene expression at day 7, as determined by means of RT-qPCR in ATDC5 cells, in the absence and presence of 10 nM BMP-2, with and without added 10 nM Noggin. The transcript of the β-actin coding gene (ACTB) was used as a housekeeping gene standard to normalize gene expression. *C*, enhancement of alkaline phosphatase activity observed at day 7 following addition of BMP-2, in the absence and presence of Noggin. Data are normalized to the total protein concentration in the supernatant. Glycosaminoglycan content and calcium deposition (*i.e.* mineralization) were probed at day 7 and 14 by (*D*) Alcian Blue and (*E*) Alizarin Red staining, respectively. A representative picture of well staining before dye extraction is shown under each corresponding bar. For all graphs, cells were grown in the medium alone (*white bars*), with 10 nM BMP-2 (*light gray bars*) or with 10 nM BMP-2 and 10 nM Noggin (*dark gray bars*). Each data represents the average value of three biological replicates. Independent t-tests were used for statistical evaluation of the data. Thus, in figures *A*–*E*, ∗∗*p* < 0.005;∗∗∗*p* < 0.001; error bars represent mean ± SD. BMP, bone morphogenetic protein; RT-qPCR, real time quantitative PCR.

Type II and type X collagens are known markers of mature and hypertrophic chondrocytes, respectively (29, 48). Hence, real time quantitative PCR (RT-qPCR) experiments (Fig. 5, *A* and *B*) were used to measure the mRNA expression of collagen type II (*Col2a1*) and collagen type X (*Col10a1*), after 7 days of cell differentiation in response to treatment with BMP-2. In agreement with previous findings (49, 50), data in Figure 5, *A* and *B* show that addition of 10 nM BMP-2 significantly promotes induction of both *Col2a1* and *Col10a1* expression. By contrast, the addition of equimolar concentration of Noggin significantly inhibits expression of the two genes.

Classical alkaline phosphatase (ALP) activity tests (49, 50) (Fig. 5*C*) showed that addition of 10 nM recombinant BMP-2 leads to a significant induction of enzymatic activity representative of the hypertrophic differentiation process. This effect, in turn, is significantly inhibited upon addition of 10 nM recombinant Noggin.

Finally, cell staining was used to emphasize the production of cartilage and bone matrices during chondrogenic differentiation. Glycosaminoglycan, a major cartilage component, was stained using Alcian Blue (Fig. 5*D*), while mineralization was quantified by Alizarin Red (Fig. 5*E*). Alcian Blue staining shows that, in the presence of 10 nM BMP-2, cartilage formation is slightly promoted at day 14, hence indicating the presence of mature chondrocytes. On the other hand, at day 14, Alizarin Red staining indicates that when BMP-2 (10 nM) is present, mineralization is substantially increased, an observation consistent with cells having acquired a predominant hypertrophic phenotype. In both cases (Fig. 5, *D* and *E*), the addition of an equimolar concentration of Noggin is seen to inhibit both cartilage and bone formation at day 14.

### Study of the N102D mutant of BMP-2

Analysis of the X-ray crystal structure of BMP-2 bound to the antagonist Noggin reveals that one-third (*i.e.* 4 out of 12) of the hydrogen bonds formed between the two proteins are contributed by the amide side chain group of residue N102 of BMP-2. Furthermore, the interaction with N102 appears to bend a segment of the N-terminal end of Noggin (*i.e.* S38 to V44) and hence facilitates its insertion into the type 1 receptor-binding site (Fig. 4*E*). In order to assess the importance of this interaction between the two proteins, we generated the N102D mutant of BMP-2. This mutation was expected to lead to the loss of at least two hydrogen bonds and also to the addition of electrostatic repulsions between the negatively charged side chain of the aspartate group and the polarized backbone carbonyls of N40 and P42 of Noggin (Fig. 4*F*).

The BMP-2 N102D mutant was efficiently produced, refolded, and purified according to the protocol developed for WT BMP-2. Thus, expression of recombinant BMP-2 N102D in *E. coli* yielded IBs, and Fig. S2*A* shows the occurrence, in the insoluble fraction mainly, of a protein with the expected apparent molecular mass (*ca*. 13 kDa). Following extraction and solubilization of IBs, refolding was achieved in one single dilution step and purification was performed on a hydrophobic interaction Source 15 ISO column. The elution profile (Fig. S2*B*) shows that BMP-2 elutes in a single sharp and symmetrical peak, suggesting the occurrence of one predominant isoform. The purity of the final BMP-2 N102D sample was checked by SDS-PAGE (Fig. S2*C*) in both reducing and nonreducing conditions and was found to be above 98%. In both cases, a major band was observed, which corresponds to an apparent molecular mass of 13 and 26 kDa, respectively, and thus suggests that BMP-2 N102D is purified as a disulphide-bonded dimer. The absence of nucleic acid contamination was shown by UV absorbance measurements (Fig. S2*D*). Like WT BMP-2, the production protocol (*i.e.* expression, refolding, and purification) yielded *ca*. 110 mg of pure, native, and fully functional (as evidenced by ALP activity measurements) BMP-2 N102D per liter of cell culture.

Analysis of the binding kinetics was performed as for WT BMP-2, using the BLI detection system (Octet HTX), and the data shown in Figure 3*B* indicated only minor differences, yielding similar values for both *k*_a_ and *k*_d_ ((2.3 ± 0.6)·10^6^ M^−1^ s^−1^ and (7 ± 0.6)·10^−4^ s^−1^, respectively) and a *K*_D_ value (0.3 ± 0.25 nM) that is identical within the error limit to that of the WT protein.

**Figure 6:**
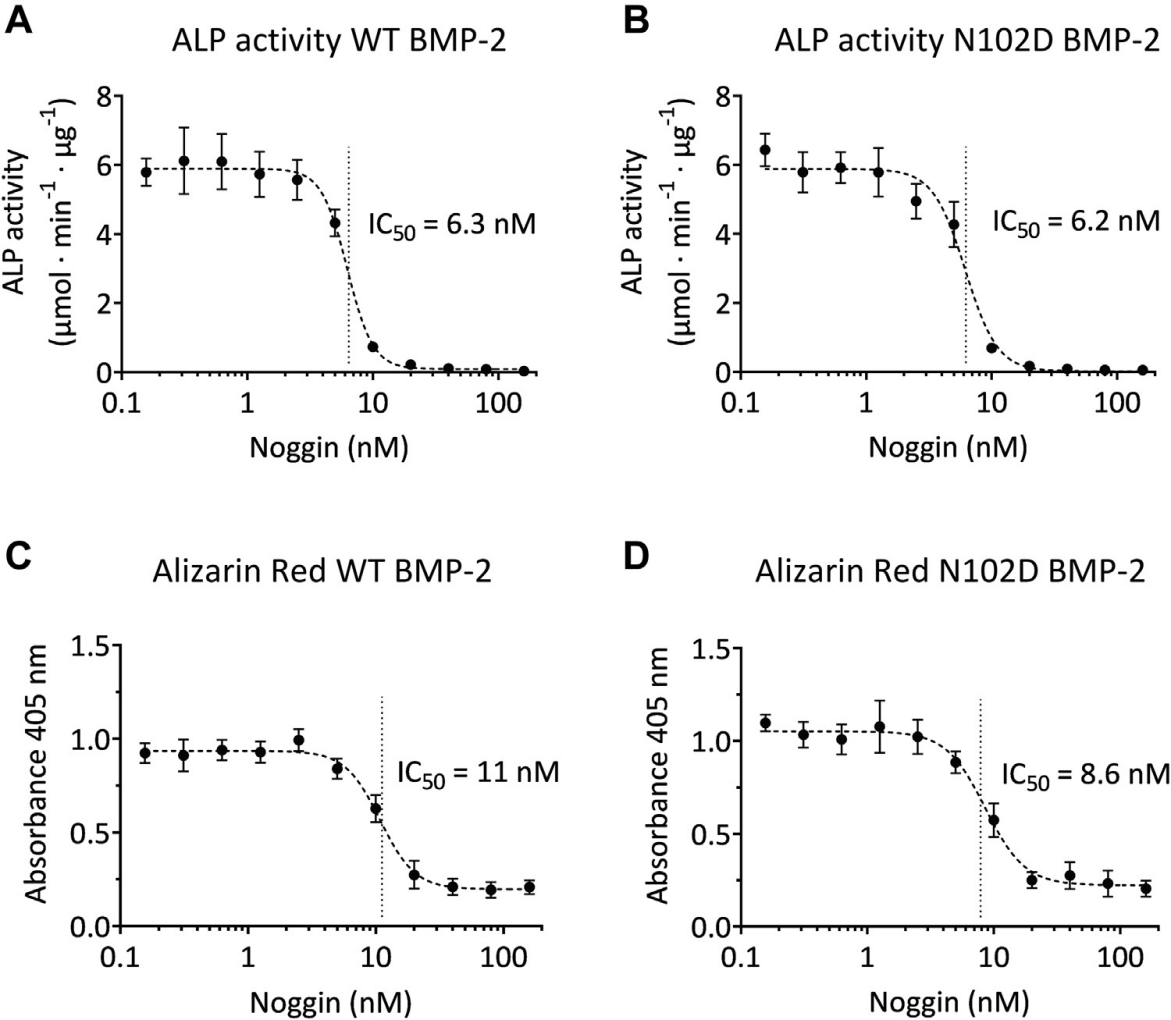
**Concentration-response curves showing the decrease in the biological activity of the WT and N102D mutant BMP-2 in the presence of increasing Noggin concentrations (plotted on a logarithmic scale).** Cells were costimulated with (*A* and *C*) 10 nM WT BMP-2 or (*B* and *D*) 10 nM mutant BMP-2, in the presence of Noggin concentrations in the 0.16 to 160 nM range. The biological activity was evaluated at day 7 on the basis (*A* and *B*) of ALP activity and (*C* and *D*) Alizarin Red staining. For all graphs, data represent the average value of six biological replicates with SD. Data were analyzed based on a sigmoidal relationship and the *dashed lines* were obtained by fitting the Hill equation to the data, using the IC_50_ values shown. For the latter, the confidence intervals are (*A*) 5.8 to 6.8, (*B*) 5.4 to 6.6, (*C*) 9.8 to 11.9, and (*D*) 7.7 to 9.6. ALP, alkaline phosphatase; BMP, bone morphogenetic protein.

In addition, we compared the sensitivity of WT and N102D BMP-2 to inhibition by Noggin. The ALP activity (Fig. 6, *A* and *B*) and calcium deposition (Fig. 6, *C* and *D*) were evaluated after the addition of 10 nM BMP-2 (WT or N102D mutant), in the presence of increasing concentration of Noggin. The ALP activity and Alizarin Red assays showed that the addition of Noggin causes a significant concentration-dependent decrease in enzymatic activity, yielding IC_50_ values that are identical, within the error limit, for both the WT and the N102D mutant BMP-2 (*i.e.* 8 ± 2 nM).

## Discussion

This study aims to better understand the relationship between BMP-2 and its antagonist Noggin, particularly in the context of articular chondrogenesis. Both BMP-2 and Noggin were overproduced in *E. coli* as IBs, as observed before (51, 52, 53, 54). This can be explained by the low solubility of the two proteins in aqueous solution, due to their complex disulphide bond network and the presence of large hydrophobic patches on their surface. To date, several different BMP-2 renaturation and purification protocols have been described (52, 53, 55, 56) but they require lengthy sample preparation steps that make them time-consuming and laborious. Here, we have developed a new and simple method to produce pure and biologically active homodimeric BMP-2. Insoluble proteins were solubilized in the presence of urea and DTT and subsequently diluted into a refolding buffer (50 mM Tris–HCl, pH 8.5). Glutathione (both reduced and oxidized) was added to promote formation of native disulphide bridges, and 1 M NaCl and 0.5 M arginine were used to minimize protein aggregation. Under these conditions, BMP-2 renaturation took 3 days at 4 °C, which compares favorably to the five (53) and fourteen (54) days of incubation required with previous protocols. This “one-step dilution” refolding is easy to perform and avoids the lengthy dialysis and concentration steps usually required (55).

For purification, refolded BMP-2 was directly loaded on a hydrophobic isopropyl matrix and then eluted by decreasing the salt concentration. This protocol takes advantage of the high salt concentration in the refolding buffer, which promotes protein adsorption on the chromatographic matrix while maintaining conditions that favor the native conformation. In comparison, previous purification strategies used multiple purification steps and required other additives (*e.g.*, urea and DMF53) (53) or involved partial unfolding of BMP-2 and buffer exchanges for final refolding. With this new protocol, 100 mg of fully active native BMP-2 could be obtained, starting from 1 l of cell culture, after only 3 days of renaturation at 4 °C and a single purification step, which is better than with previous protocols.

The quality of the protein reagents used in this study was analyzed according to the ARBRE-MOBIEU guidelines (35, 36). Thus, minimal QC tests showed that BMP-2 and Noggin were pure, homogeneous in terms of size distribution, and that their identity and integrity were beyond doubt. Moreover, BMP-2 and Noggin have been characterized using optical methods. With both proteins, fluorescence spectra indicated stable tertiary contacts, while CD spectra showed well-organized secondary structure elements. In both cases, however, analyses of the CD spectra led to significant underestimation of the α-helix content compared to the X-ray crystal structure analysis. Thus, calculations from CD yielded (4 ± 1.5) % and (6 ± 3) % helical content for BMP-2 and Noggin, respectively, whereas X-ray structures of the two proteins gave *ca*. 15% and 18%, respectively. The possibility of significant conformational changes upon complex formation can be ruled out for BMP-2, as its structure when bound to the antagonist is indistinguishable from that reported for the cytokine alone in solution (10). As for Noggin, however, no structure of the unbound molecule is currently available and therefore a conformational change cannot be excluded. Nevertheless, estimation of the secondary structure content of a protein from its CD spectrum is empirical, due to the lack of a unique solution for the decomposition of the spectrum and also to the various assumptions involved (57). In particular, the analysis rests on the idea that only peptide chromophores determine the far-UV CD spectrum and contributions from any other chromophores of the protein can be neglected. Little is known, however, about the higher-energy transitions in disulphide bonds (58), and the many bonds of this kind in the two proteins studied in this work might well contribute to their far-UV CD spectrum. Finally, the BMP-2 spectrum (Fig. 1*H*) is very peculiar and differs from the reference spectra used for secondary structure calculations; it could therefore lead to a bias in the secondary structure content estimates. Nevertheless, the spectral signatures of BMP-2 and Noggin are very similar to those previously recorded (21, 38). This finding shows that CD spectra can be used for a rapid identification of the correct folding of these proteins, which is particularly useful for assessing lot-to-lot consistency, as well as protein stability over time.

The kinetics of binding were analyzed by BLI and data indicated that Noggin interacts with BMP-2 according to a 1:1 reaction stoichiometry, with a dissociation constant (*K*_D_) in the subnanomolar range (*ca*. 0.4 nM). This value, which constitutes an indirect validation of the correct folding of the two proteins, is consistent with the *K*_D_ (0.6 nM) obtained previously with SPR (40) and confirms the observation that Noggin binds BMP-2 with high affinity. For comparison, Gremlin-1 and Gremlin-2 antagonists have been shown to bind BMP-2 and GDF-5 (BMP-14), respectively, with a dissociation constant of *ca.* 9 nM (21, 43).

Although BMP-2 is known to play an important role during articular chondrogenesis, its complex autocrine and paracrine signaling pathways (59) hamper its clinical potential and prompts the need for a better understanding of its activity and regulation mechanisms (25, 60). In this work, we monitored the differentiation of ATDC5 cells upon addition of BMP-2, in the presence or absence of Noggin. Reproducible *in vitro* cellular assays confirmed the biological activity of both ligands. Thus, data from RT-qPCR and matrix staining showed that addition of BMP-2 induces the differentiation of ATDC5 cells into mature chondrocytes, as indicated by an increase in both *Col2a1* gene expression and cartilage matrix synthesis. Likewise, these experiments also demonstrated that BMP-2 promotes the differentiation of mature chondrocytes into hypertrophic chondrocytes, as indicated by both the rise in the expression of *Col10a1* and the mineralization of the cartilage matrix. Finally, when BMP-2 was incubated in the presence of an equimolar amount of Noggin, no significant cell differentiation was detectable either after 7 (ALP activity and RT-qPCR) or 14 (cell staining) days of incubation. Overall, these results support previous conclusions (59) that BMP-2 induce the differentiation of mature chondrocytes into hypertrophic chondrocytes and also provide clear evidence for its high sensitivity to Noggin inhibition. They show obvious limitations to the use of BMP-2 in cartilage regeneration.

**Figure 7:**
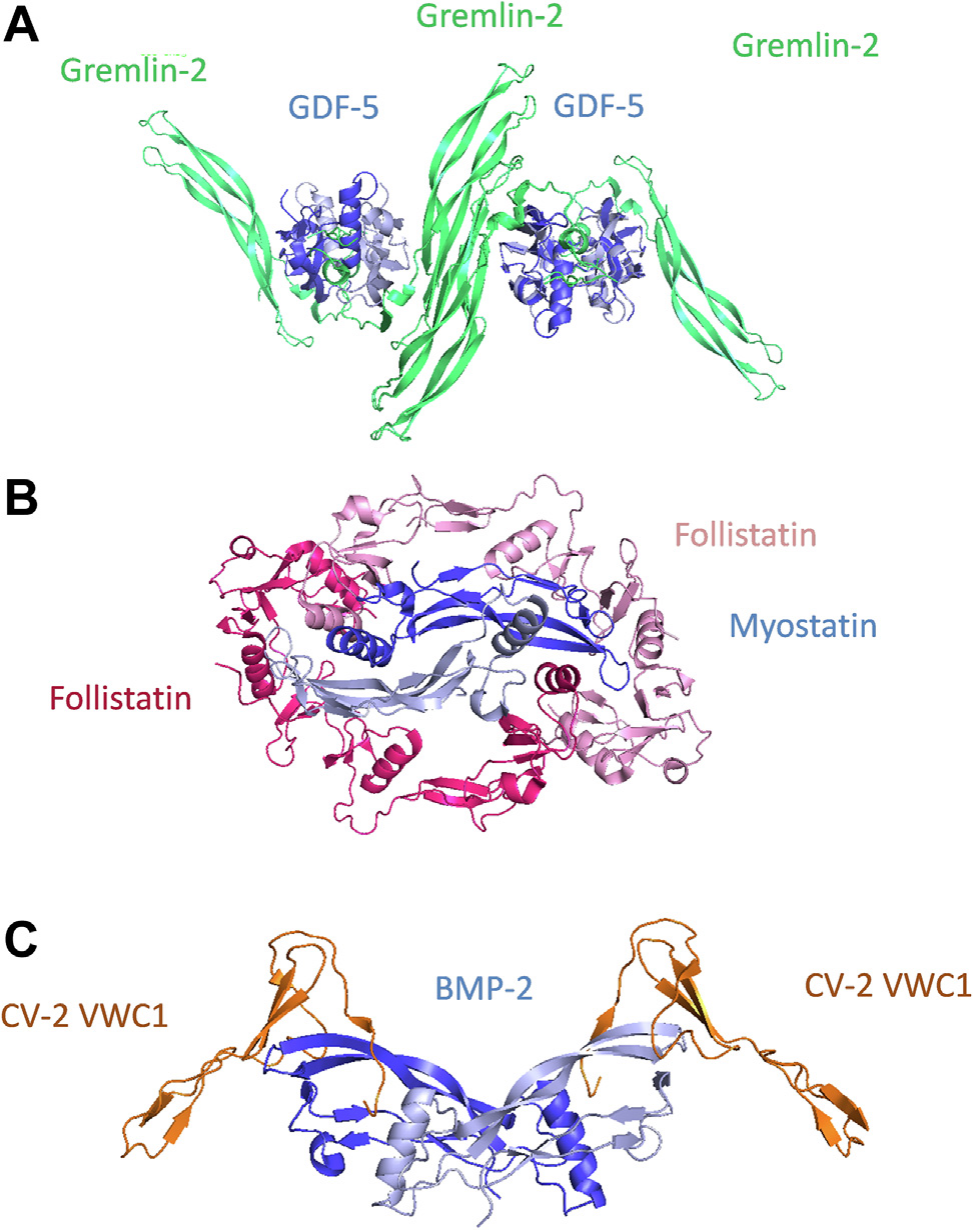
**Diversity of structures and inhibition modes of BMP antagonists.***A*, X-ray crystal structure of GDF5 (BMP-14) in complex with Gremlin-2 (Protein Data Bank code 5HK5) (43). GDF5 is shown in *dark* and *light blue* to distinguish between the two monomers, while Gremlin is shown in *green*. *B*, X-ray structure of Myostatin in complex with two molecules of Follistatin (Protein Data Bank code 3HH2) (44). Myostatin is in *dark* and *light blue* to distinguish between the two monomers, while Follistatin molecules are shown in *dark* and *light pink* to distinguish between the two molecules. *C*, X-ray structure of BMP-2 in complex with two CV-2 VWC1 molecules (Protein Data Bank code 3BK3) (45). BMP-2 is in *dark* and *light blue* to distinguish between the two monomers, while the VWC1 domain of CV-2 is represented in *orange*. BMP, bone morphogenetic protein; CV-2, Crossveinless 2; VWC1, Von Willebrand factor type C domain 1.

To investigate in greater detail the structural basis for the efficacy of Noggin as an inhibitor of BMP signaling in cell chondrogenic differentiation, the complex with BMP-2 was crystallized and the structure solved at 3.1 Å by using X-ray diffraction (Fig. 4). It suggests a simple 1:1 inhibition mechanism, as observed previously with the BMP-7:Noggin complex (22), where one single antagonist molecule binds to one BMP molecule. Alignment of the BMP-2 and BMP-7 sequences and structural comparison of the complexes they form with Noggin reveal two highly similar binding interfaces. A difference is observed, however, in the linker region, where residues N102 and Q104 of BMP-2 are replaced by K127 and R129, respectively. These two substitutions, which are associated with the loss of three hydrogen bonds in the BMP-7:Noggin interface, however, do not result in a substantial difference in the dissociation constants for binding of the antagonist to BMP-2 and BMP-7 (0.6 and 0.2 nM, respectively, as measured with SPR (40)). Among the antagonists whose structure of the complex formed with a TGF-β is known, the simple 1:1 mode of interaction is unique to Noggin. Antagonists differ significantly in terms of size, sequence, and fold and are characterized by a broad structural and functional diversity in their modes of inhibition. Thus, Gremlin-1 and Gremlin-2 (Fig. 7*A*) have been shown to sequester BMP molecules in large, aggregate-like, oligomeric species *in vitro* (21, 43). In the case of Follistatin (Fig. 7*B*), two molecules were found to cover the entire circumference of the growth factor Myostatin (GDF-8) (44). Finally, the crystal structure of the Von Willebrand factor type C domain 1 of Crossveinless 2 (CV-2) (Fig. 7*C*), a BMP modulator protein (it can either enhance or inhibit BMP activity) bound to BMP-2, revealed that two CV-2 molecules bind one BMP-2 molecule through their canonical Von Willebrand factor type C domain 1 (45). Despite these marked structural differences, all antagonists inhibit BMP signaling on the same molecular basis, namely by docking to their type 1 and 2 receptor-binding sites (Fig. 7).

With BMP-2, structural examination of the complexes formed with Noggin and with its receptors (12, 61) reveals that the binding interface with the antagonist can be divided into three main contact regions (Fig. 4). The first is a short hydrophobic segment (P35A36P37) of Noggin (Fig. 4*C*), where the pyrrolidine ring of P35 fits into a large hydrophobic pocket of BMP-2, which is otherwise occupied by the benzyl group of the conserved F85 of the type 1 receptor. The second contact region (Fig. 4*D*) consists of a large concave hydrophobic patch in the core of Noggin, which masks the convex, hydrophobic-binding site of BMP-2 for the type 2 receptor. Finally, a Noggin linker segment forms an extensive network of polar bonds with the region between the BMP-2 type 1 and 2 receptor-binding sites (Fig. 4*E*). In this region of the BMP-2 structure, the N102 residue seems to play a key role in stabilizing the complex because it is involved in four hydrogen bonds (*i.e.* three with the amide side-chain group and one with the backbone carbonyl) with Noggin. Moreover, it appears to reinforce the bend of the flexible N-terminal end of Noggin and hence facilitate its insertion into the binding site of the BMP-2 type 1 receptor.

**Figure 8:**
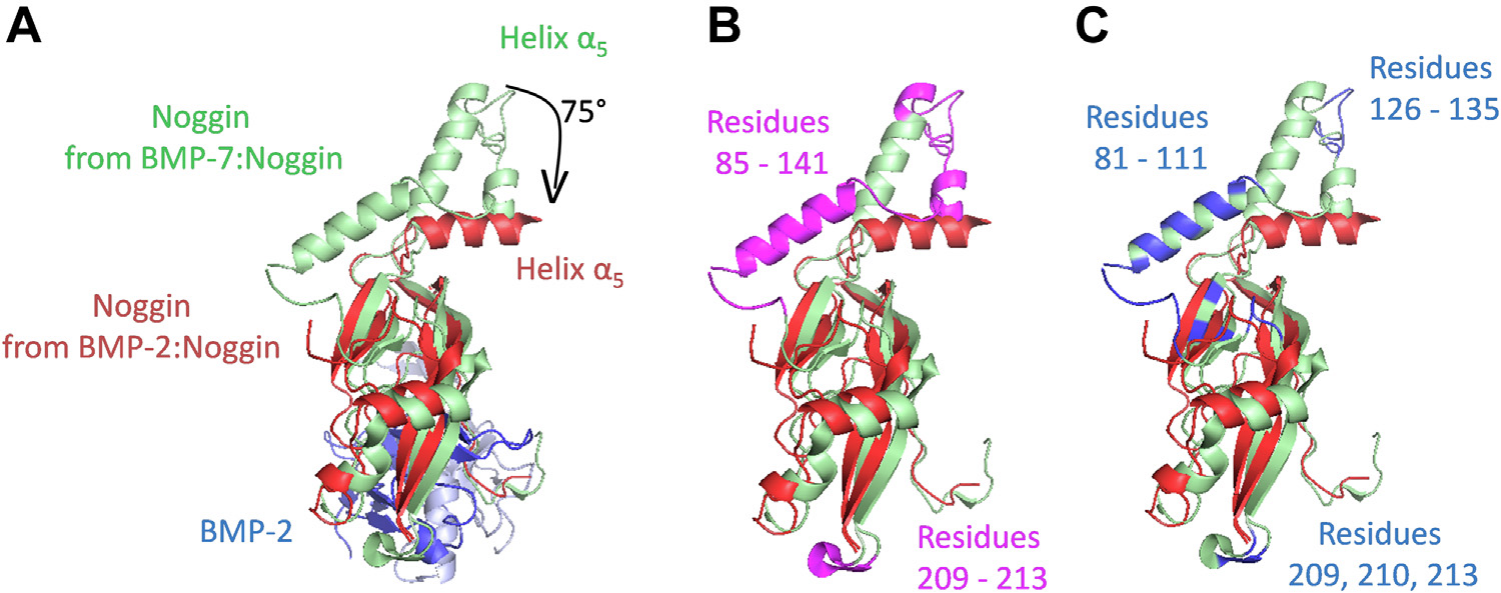
**Overlay of Noggin structures bound to BMP-2 (****7AGO****; this work) and BMP-7 (****1M4U****)** (22)**.***A*, the structure of the BMP-2:Noggin complex obtained in this work is shown, with the two proteins colored blue and red, respectively. In addition, the structure of Noggin bound to BMP-7 (the latter has been omitted from the picture for the sake of clarity) is shown in *green*. *B*, Noggin residues 84-141 and 209-213 that were not resolved in the structure of the complex with BMP-2 (this work) are highlighted in *magenta*. *C*, Noggin residues (81–111, 126–135, 209, 210, and 213) that were stabilized by artefactual crystal packing interactions are highlighted in *blue*. Figures were generated using the open-source molecular graphics system PyMOL (The PyMOL Molecular Graphics System, Version 4.5.0., Schrödinger, LLC). BMP, bone morphogenetic protein.

To examine the importance of residue 102 of BMP-2 in the context of its interaction with Noggin, the N102D mutant was produced and characterized on the same bases than the WT protein. Substitution of an amide with a carboxylic group was expected to lead to both the loss of two hydrogen bonds and the introduction of an electrostatic repulsion with the backbone carbonyl groups of the N40 and P42 residues of Noggin (Fig. 4, *E* and *F*). In addition, the choice of the mutation was guided by the observation that Activin A, another member of the TGF-β/BMP ligand superfamily (62), known to be resistant to Noggin inhibition (31, 63), displays an aspartate residue in an equivalent position (64). However, a comparison of the two BMP-2 molecules, particularly their interaction with Noggin, revealed no significant differences. This observation could be interpreted as the result of the intrinsic flexibility and dynamics of the Noggin molecule, which would allow subtle conformational changes in the protein to accommodate the mutation. Indeed, the X-ray diffraction data obtained in this work indicate that this protein molecule is more flexible than previously described. In particular, the comparison of the structure of the BMP-2:Noggin complex with its BMP-7:Noggin (22) counterparts suggests that the homodimerization interface of Noggin can adopt different conformations. Thus, in the BMP-2:Noggin complex, the α5 helix of Noggin is offset by approximately 75 degrees relative to BMP-7:Noggin (Fig. 8A). Moreover, residues from 84 to 141, which are part of the dimerization interface, and residues from 209 to 213, which form a loop connecting strands β3 and β4, are not defined in our structure, probably as a result of their high flexibility (Fig. 8*B*). Although this contrasts with X-ray data obtained with the BMP-7:Noggin complex, analysis of the crystal structure of the latter with the PDBePISA server suggests that the Noggin homodimerization interface may be specifically stabilized through crystal packing artefacts (Fig. 8*C*). These results testify to the great flexibility and dynamics of Noggin. This is consistent with data obtained with other BMP antagonists. For example, Gremlin and CV-2 (see above) exhibit a flexible N-terminus end, which protrudes into the type 1 receptor-binding site (21, 43, 45), while Follistatin likely changes conformation to sequester different ligands (44). Taken together, the data obtained with the BMP-2 N102D mutant suggest that Noggin flexibility is a parameter to be considered for the design of BMP-2 variants with reduced affinity for this antagonist (and probably for any other antagonist as well). Nevertheless, given the large binding interface between the two proteins, destabilization of the sole contact region centered around the N102 residue of BMP-2 may well be insufficient to significantly reduce the binding affinity between the two proteins. Thus, while a single point mutation was found to have too limited effects, and the same could likely be observed for any single point mutation at the binding interface, combining multiple synergistic mutations could be an effective strategy for the selection of BMP-2 mutants with a greatly reduced affinity for Noggin. Other mutants are currently being studied in our group to validate these conclusions.

## Experimental procedures

### Production of both WT BMP-2 and N102D mutant

The gene coding for the mature form of BMP-2 (residues 283–396, UniProt #P12643) was optimized for both codon usage and mRNA secondary structure, with the aim of high level of heterologous expression in *E. coli*. The complementary DNA (cDNA) insert was chemically synthesized and subcloned into a pET-17(b) vector by GeneCust Services, and authenticity of the expression vector was verified by Sanger sequencing. The purified plasmid was inserted by transformation into *E. coli* BL21 (DE3) cells and a colony was selected on an LB agar plate supplemented with 100 μg mL^-1^ ampicillin. A 20 ml starter culture was grown overnight at 37 °C with shaking at 250 rpm in LB medium supplemented with ampicillin (100 μg mL^−1^). Ten milliliters of this culture were used to inoculate 1 l of TB medium supplemented with ampicillin (100 μg mL^−1^), grown at 37 °C with shaking at 250 rpm until the absorbance at 600 nm (Abs_600nm_) reached a value comprised between 0.4 and 0.6, then induction was carried out by the addition of 1 mM IPTG and the bacteria were grown for a further 16 h at 18 °C. Finally, cells were collected by centrifugation at 5000*g* for 15 min at 4 °C. Proteins were analyzed following separation on 4 to 20% SDS-PAGE and staining with Coomassie blue or western blot using a mouse antibody with specificity for BMP-2 (provided by the CER Group).

BMP-2 was expressed as IBs, which were solubilized before refolding to the native active form. Two grams of cell pellet, harvested from 200 ml of cell culture, were homogenized in 25 ml lysis buffer (20 mM Tris–HCl, pH 8, with 5 mM EDTA) and sonicated on ice. The lysate was then cleared by centrifugation (30 min, 15,000 g, 4 °C) and insoluble materials containing the IBs were recovered. This was sequentially resuspended in 25 ml 100 mM Tris–HCl, pH 8, with 1% Trition X-114, incubated for 10 min at 56 °C, and finally centrifuged at 30,000 g for 15 min at 20 °C. This series of three steps was repeated three times to remove lipids, membrane proteins, and also endotoxins (65). Next, IBs were solubilized overnight at 4 °C in 30 ml solubilization buffer (50 mM Tris–HCl, pH 8, 1 mM EDTA, 10 mM DTT and 8 M urea), and the supernatant was then recovered by centrifugation at 15,000*g* for 15 min at 4 °C. At this stage, total protein concentration in the supernatant was estimated by absorbance measurement at 280 nm and, *ca.* 100 mg of solubilized IBs were diluted to 0.1 mg mL^-1^ in 1l of refolding buffer (50 mM Tris–HCl, pH 8.5, 1 M NaCl, 5 mM EDTA, 1.5 mM GSSG, 3 mM GSH, and 0.5 M arginine) and incubated for 3 days at 15 °C under slow stirring. The 33-fold dilution factor resulted in residual concentrations of urea and DTT in the refolding buffer of about 240 mM and 0.3 mM, respectively. This procedure led to BMP-2 refolding, which was subsequently centrifuged (10 min, 10,000*g*, 4 °C) and filtered on 0.45 μm to remove misfolded and precipitated proteins. Purification of refolded BMP-2 was then carried out on a 20 ml Source 15 ISO (isopropyl) column (Cytiva), equilibrated with buffer A (50 mM Tris–HCl, pH 7, 3M NaCl, 5 mM EDTA, and 0.5 M arginine). Following sample loading, the column was extensively washed to remove unbound proteins, and BMP-2 was eluted with a linear gradient of buffer B (50 mM Tris–HCl, pH 7, 5 mM EDTA, and 0.5 M arginine). The fractions containing the purified BMP-2 were pooled and dialyzed against 100 volumes of 50 mM Tris–HCl, pH 7, 5 mM EDTA, and 0.5 M arginine. Final protein concentration was determined through absorbance measurements performed at 280 nm in a 1-cm pathlength cell, using the molar extinction coefficient estimated from the amino acid composition of BMP-2 (37650 M^−1^·cm^−1^) (66). The samples were aliquoted, filtered on 0.22 μm, and stored at −20 °C.

### Production of Noggin

After computing both the codon usage and mRNA secondary structure for an efficient expression in *E. coli*, the Noggin sequence (residues 28–232, UniProt #Q13253) was inserted into a pET-17(b) vector by GeneCust Services and authenticity of the expression vector was verified by Sanger sequencing. The recombinant plasmid was then transferred into *E. coli* BL21 (DE3) for protein expression, and successfully transformed cells were selected on LB agar plate with 100 μg mL^−1^ of ampicillin. A 20 ml starter culture was grown overnight at 37 °C with shaking at 250 rpm in LB medium supplemented with ampicillin (100 μg mL^−1^). After, 10 ml of this were used to inoculate 1l of TB with ampicillin (100 μg mL^−1^). Culture was grown at 37 °C with shaking at 250 rpm to achieve an Abs_600nm_ of approximately 0.5, then Noggin production was induced by the addition of 1 mM IPTG and culture was incubated for a further 16 h at 18 °C. Cells were finally collected by centrifugation at 5000*g* for 15 min at 4 °C. Proteins were analyzed following separation on 4 to 20% SDS-PAGE and staining with Coomassie blue or western blot using a mouse antibody with specificity for Noggin (MyBioSource).

Noggin was expressed insolubly and subsequently refolded to an active form as described (22). Briefly, 6 g of cell pellet, harvested from 200 ml of cell culture, were resuspended in 25 ml of lysis buffer (20 mM Tris–HCl, pH 8, 5 mM EDTA, 20 mM DTT, and 1% Triton X-100) and lysed by sonication on ice. After centrifugation (15,000*g*, 30 min, 4 °C), the pellet containing Noggin IBs was recovered. Endotoxins, membrane proteins, and lipids were removed from the pellet by a series of three steps, repeated three times (65): first, bacteria were resuspended in 100 mM Tris–HCl pH 8 with 1% Trition X-114, incubated for 10 min at 56 °C, and finally centrifuged at 30,000*g* for 15 min at 20 °C. After this, washed IBs were solubilized overnight at 4 °C in 30 ml of solubilization buffer 1 (50 mM Tris–HCl, pH 8, 1 mM EDTA, 10 mM DTT, and 6 M guanidinium chloride). After centrifugation (15, 000*g*, 15 min, 4 °C), the supernatant was diafiltered with Amicon (10 kDa cut off, Merck) against five volumes of solubilization buffer 2 (50 mM sodium acetate, pH 4.5, 1 mM DTT, and 6M urea). Total protein concentration was estimated through absorbance measurements performed at 280 nm in a 1-cm pathlength cell. To refold Noggin to active form, 200 mg of solubilized IBs were diluted to 0.5 mg mL^−1^ in 400 ml of refolding buffer (50 mM Tris–HCl, pH 8, 1 mM EDTA, 0.2 mM GSSG, 2 mM GSH, and 1.5 M guanidinium chloride) and incubated for 3 days at 4 °C under slow stirring. The Noggin solution was further dialyzed twice against 10 volumes of 50 mM sodium citrate, pH 6.8, 150 mM NaCl, 1 mM magnesium acetate, 20% glycerol, and 0.02% CHAPS. Misfolded Noggin precipitated and was subsequently removed by centrifugation at 10,000*g* for 10 min at 4 °C. Finally, refolded Noggin was purified on a 5 ml HiTrap SP Sepharose FF column (Cytiva), equilibrated with buffer A (50 mM sodium citrate, pH 6.8, 150 mM NaCl, 1 mM magnesium acetate, 20% glycerol, and 0.02% CHAPS). After washes to remove unbound proteins, Noggin was eluted with a linear gradient of buffer B (50 mM sodium citrate, pH 6.8, 1.15 M NaCl, 1 mM magnesium acetate, 20% glycerol, and 0.02% CHAPS). The fractions containing the purified Noggin were pooled and dialyzed overnight against 100 volumes of storage buffer (50 mM sodium citrate, pH 6.8, 150 mM NaCl, 1 mM magnesium acetate, 20% glycerol, and 0.02% CHAPS). Protein concentration was determined through absorbance measurements performed at 280 nm in a 1-cm pathlength cell, using the molar extinction coefficient estimated from the amino acid composition of Noggin (81,900 M^−1.^cm^−1^) (66). Samples were aliquoted, filtered on 0.22 μm, and stored at −20 °C.

### Size-exclusion chromatography with multi-angle light scattering

Experiments were performed at room temperature (*ca*. 20 °C) using an LC-20 Prominence BioInert HPLC system (Shimadzu), coupled to a Wyatt miniDAWN TREOS-II MALS instrument, a Refractive Index Detector RID-20A, and a UV-VIS Detectors SPD-20A (Shimadzu). For chromatographic separation, samples were loaded on a Superdex 200 Increase 10/300 Gl (Cytiva) equilibrated in the running buffer A for BMP-2 (50 mM Tris–HCl, pH 7, 5 mM EDTA, and 0.5 M arginine) or in the running buffer B for Noggin (50 mM sodium citrate, pH 6.8, 150 mM NaCl, 1 mM magnesium acetate, 20% glycerol, and 0.02% CHAPS). Data were analyzed with ASTRA software (Wyatt Technology) and molecular mass was calculated using a Debye fit model. Note that the apparent molecular mass values obtained with both Noggin and the BMP-2:Noggin complex appeared 16 and 11% lower than expected, respectively. A similar underestimation (18%) was observed with bovine serum albumin, used as a standard, in the presence of CHAPS and this was tentatively attributed to an effect of the detergent on the IR signal.

### Mass spectrometry analysis and data treatment

Purified proteins were characterized by ultra-high performance liquid chromatography coupled to high resolution mass spectrometry (UHPLC-HRMS) using intact protein analysis. Proteins were first separated by reverse-phase liquid chromatography with an ACQUITY UPLC Protein BEH C4 Column (300 Å, 1.7 μm, 2.1 mm X 50 mm) (Waters) and using a water/acetonitrile gradient containing 0.1 % of formic acid. Eluted compounds were ionized by an electrospray ionization source in positive mode. The data were then acquired in MS mode with a 1 s scan time within the 400 to 5000 m/z mass range with a Xevo G2-XS QTof quadrupole time-of-flight system (Waters). Acquired data were processed using UNIFI software (Waters) and intact protein analysis type with MaxEnt1 algorithm for charge envelope deconvolution.

### Absorbance measurements

Absorbance spectra were recorded at 25 °C using a Jasco V-630 spectrophotometer. Each spectrum was taken as the average of two individual acquisitions and corrected for the contribution of the buffer solution.

### Fluorescence measurements

Intrinsic fluorescence emission spectra were acquired at 25 °C with a Varian Cary Eclipse spectrofluorimeter equipped with a Peltier-controlled cell holder, using 1 cm pathlength quartz cuvettes, and with protein concentrations of *ca.* 0.1 mg mL^−1^. BMP-2 spectra were measured in 50 mM Tris–HCl, pH 7, in the presence of 5 mM EDTA and 0.5 M arginine, whereas Noggin spectra were acquired in 50 mM sodium citrate, pH 6.8, 150 mM NaCl, 1 mM magnesium acetate, 20% glycerol, and 0.02% CHAPS. Excitation and emission slit widths were 5 nm, and the scan speed was 600 nm*·*min^−1^. Each spectrum is the average of five individual acquisitions and was corrected for the contribution of the buffer solution.

### Circular dichroism measurements

Far-UV circular dichroism spectra of BMP-2 were recorded at 25 °C on a Jasco J-810 spectropolarimeter in 20 mM sodium phosphate buffer, pH 7, using a 1 mm pathlength quartz Suprasil cell (Hellma), with protein concentrations of *ca*. 0.1 mg mL^−1^. Four scans (10 nm/min, 1 nm bandwidth, 0.1 nm data pitch, and 4 s DIT) were averaged, base lines were subtracted, and no smoothing was applied. Data are presented as the molar residue ellipticity ([Ɵ]_MRW_) calculated using the molar concentration of protein and the number of residues. With Noggin, synchrotron radiation CD spectra were recorded at 25 °C on the DISCO beamline of synchrotron SOLEIL in 50 mM sodium citrate buffer, pH 6.8, with 150 mM NaCl, 1 mM magnesium acetate, 20% glycerol, and 0.02% CHAPS, using a 0.1 mm pathlength quartz Suprasil cell (Hellma), with a protein concentration of 0.88 mg mL^−1^. Each synchrotron radiation CD spectrum represents the average of four individual scans (1 nm/min, 1 nm bandwidth, 1 nm data pitch, and 1.2 s integration time) and the contribution of the buffer was subtracted from far-UV CD spectrum of the sample.

Secondary structure analyses using the CDSSTR (67, 68) and CONTINLL (68, 69, 70) algorithms were performed on the CD data with the CDPro software package (68). The results from the two algorithms were averaged and the SDs were calculated.

### Bio-layer interferometry

BLI experiments were performed at Robotein (www.robotein.ulg.ac.be), using an Octet HTX instrument (FortéBio, Sartorius). For binding assays, biotinylated Noggin (4 μg mL^−1^) was immobilized on streptavidin-coated biosensors (Sartorius) and concentrations of BMP-2 were in the range of 1 to 22.8 nM. Notably, optimization of the immobilized concentrations of biotinylated Noggin onto the biosensors was performed in order to minimize potential “avidity effects” that can be observed due to high ligand density immobilized on the biosensors surface when using a bivalent analyte (in this case dimeric BMP-2). This phenomenon can lead to an underestimation of the *k*_d_ value and therefore an overestimation of the binding affinity. All experiments were carried out at 30 °C in kinetic buffer (50 mM sodium citrate, pH 6.8, 150 mM NaCl, 1 mM magnesium acetate, and 0.02% CHAPS). After loading with biotinylated Noggin, the biosensors were saturated in a biocytin solution (10 μg mL^−1^) for 120 s and then dipped into the kinetic buffer solution for 300 s to monitor the baseline. Association and dissociation time were respectively set to 180 s and 300 s. Data were corrected for the contribution of the buffer solution and binding affinities were calculated using the Octet software version 8.0, according to a 1:1 interaction model. This experiment was conducted three times using independent protein dilutions.

### Formation of the BMP-2:Noggin complex and crystallization

The two proteins were mixed at an equimolar ratio in 20 mM Tris–HCl buffer, pH 7.4, with 700 mM NaCl and 1.8% CHAPS. The resulting complex was purified by SEC using a Superdex 200 10/300 Gl (Cytiva) column. Crystallization assays were carried out at 20 °C using the hanging drop vapor diffusion method by mixing 2 μl of BMP-2:Noggin (15 mg mL^−1^) with 2 μl of a crystallization solution composed of 1.62 M NaH2PO4, 0.18 M K2HPO4, pH 5.6. Rod-shaped crystals of space group P6122 were obtained and transferred in a cryoprotectant solution (45% (v/v) glycerol and 1.8 M ammonium sulfate) before freezing in liquid nitrogen.

### Data collection and structure determination

The diffraction data were collected on the Proxima1 beamline of the Soleil synchrotron. The data were indexed, integrated, and scaled using XDS (71). The structure of the BMP-2:Noggin complex was solved by molecular replacement with Phaser (72), using the structure of Noggin bound to BMP-7 as a model (Protein Data Bank code: 1M4U) (22). The asymmetric unit contains one BMP-2 monomer in interaction with one Noggin monomer. The refinement and model building cycles were performed with phenix.refine (73) and Coot (74), respectively. A summary of X-ray diffraction data and refinement statistics is given in Table S1.

### Cell culture

Mouse chondrogenic ATDC5 cells (Sigma Aldrich) were grown in a proliferation medium (DMEM/F-12, GlutaMAX supplement (Thermo Fisher Scientific), 5% fetal bovine serum (Biowest), and 0.5 % Pen-Strep (Invitrogen)), under a humidified atmosphere at 37 °C, 5% CO_2_. Upon reaching confluency, cells were harvested and plated at 6400 cells·cm-2. Chondrogenic differentiation was induced by replacing the proliferation medium with a differentiation medium (proliferation medium supplemented with 10 μg mL^−1^ insulin (Sigma-Aldrich), 10 μg·mL^−1^ transferrin (Sigma-Aldrich), 30 nM sodium selenite (Sigma-Aldrich), 50 μg mL^−1^ ascorbic acid (Sigma-Aldrich)) and by adding BMP-2 and Noggin at various concentrations. In these experiments, the differentiation medium was refreshed every 3 days.

### ALP activity assays

Enzymatic activity of ALP in ATDC5 cell cultures was measured with Alkaline Phosphatase Assay Kit (Abcam). Cells were harvested, washed in PBS, and lysed in ALP Assay buffer. Following centrifugation at 6500*g* for 15 min at 4 °C, ALP activity was measured in the supernatant by monitoring the dephosphorylation of p-nitrophenyl phosphate into p-nitrophenol, through changes in absorbance measured at 405 nm, with the help of a micro-plate reader (SpectraMax 190, Molecular Device). Total protein concentration in cell cultures was determined by BCA Protein assay (Thermo Fisher Scientific). A standard curve was used to determine the absolute amount of ALP-generated nitrophenol over time. Each value was the average of three individual acquisitions and was normalized to the total protein content. Enzyme activity was calculated as the number of μmol of nitrophenol formed in 1 min per 1 μg of protein (*i.e.* μmol·min^−1^ μg^−1^).

### RNA isolation and RT-qPCR

RNA was isolated using High Pure RNA Isolation Kit (Roche) according to the manufacturer’s instructions. The amount and purity of RNA were assessed by measuring the absorbance at 260 nm (Abs_260_) and the Abs_260_/Abs_280_ ratio, respectively, using a spectrophotometer (Biochrom). RNA was diluted to 100 ng·mL^−1^ using DNase/RNase free water and then stored at −80 °C. RNA was reverse transcribed into cDNA using qScript cDNA Synthesis Kit (Quanta bio) following the manufacturer’s instructions. For RT-qPCR, cDNA were diluted three times in DNase/RNase free water and mixed with FastStart Essential DNA Green Master (Roche) and 300 nM forward and reverse primers. Primer sequences are given in Table S2.

Amplification curves were obtained using a LightCycler 96 Real-Time PCR Cycler (Roche), using a 3-steps protocol: denaturation for 600 s at 95 °C, followed by 50 cycles of amplification (10 s at 95 °C, 15 s at 60 °C, and 17 s at 72 °C) and then a melting curve (10 s at 95 °C, 60 s at 65 °C, and 1 s at 97 °C). Each measurement was the average of three individual acquisitions and the relative quantification of each gene was obtained after normalization against the housekeeping gene *β-actin*.

### Collagen staining

Cells were washed two times with PBS, fixed with 4% paraformaldehyde in PBS for 10 min at room temperature, and then washed three times with distilled water to remove paraformaldehyde. To stain proteoglycans and calcium deposition, cells were incubated for 30 min, at room temperature, with 1% (m/v) Alcian Blue (Agilent) and with 40 mM Alizarin Red S, pH 4.2 (Thermo Fisher Scientific), respectively. Then, cells were washed three times with distilled water to remove unbound dye and allowed to air dry. Pictures were acquired with a ZEISS Axio Vert.A1 microscope. After this, Alcian Blue and Alizarin Red were extracted by incubation for 1 h on a plate shaker in 6 M Guanidine-HCl and in 10% acetic acid, respectively. Extracted dyes were finally quantified through absorbance measurements at 645 and 405 nm, for Alcian Blue and Alizarin Red, respectively, using a micro-plate reader (SpectraMax 190, Molecular Device). For comparison between WT and N102D BMP-2, the Alizarin Red staining assay was carried out with a cell culture medium supplemented with 3 mM Na_2_HPO_4_ (Sigma-Aldrich), because the presence of phosphate facilitates mineralization and hence gives faster response during differentiation.

### Statistics

Unless otherwise stated, errors are reported as SDs throughout. Threshold for statistical significance was 0.05. In figures, ∗∗ to *p* ≤ 0.01, ∗∗∗ to *p* ≤ 0.001. In text and figures, data distributions are reported as mean ± SD. In Figure 5, statistical evaluation was carried out with independent t-tests.

## Data availability

The electron density map and structural model of the BMP-2:Noggin complex developed in this study are available in the Protein Data Bank (code 7AG0).

## Supporting information

This article contains supporting information.

## Conflict of interest

F. K. is research associate of the FRS-FNRS (Brussels, Belgium). The authors declare that they have no conflicts of interest with the contents of this article.
