# Supplementary material for: Structural analysis of the interaction between human cytokine BMP-2 and the antagonist Noggin reveals molecular details of cell chondrogenesis inhibition
Source: J Biol Chem. 2023 Jan 13;299(2):102892. doi: 10.1016/j.jbc.2023.102892 (PMC9929448; doi:10.1016/j.jbc.2023.102892)
Supplement: Supporting information [file mmc1.docx]

**Functional and structural characterization of human Bone Morphogenetic Protein-2 and the antagonist Noggin**

Charly Robert, Frédéric Kerff, Fabrice Bouillenne, Maxime Gavage, Marylène Vandevenne, Patrice Filée, and André Matagne.

**Supporting informations**

**Table S1**.

**Table S2.**

**Figure S1.**

**Figure S2.**

**Table S1**. Crystallographic data and refinement statistics (molecular replacement) for the BMP-2:Noggin complex. Values in parenthesis refer to the highest resolution shell.

| **Data Collection** | |
| --- | --- |
| Wavelength (Å) | 0.978565 |
| Space group | P6_1_22 |
| a, b, c (Å) | 103.3, 103.3, 170.1 |
| α, β, γ (°) | 90, 90, 120 |
| Resolution range (Å) | 49.43 - 3.1 (3.18 - 3.1) |
| Rmerge (%) | 53.1 (556) |
| <I>/<σI> | 9.5 (0.69) |
| Completeness (%) | 99.9 (99.6) |
| Redundancy | 56.6 (49.3) |
| CC [1/2] | 97,7 (32) |
| **Refinement** | |
| Resolution range (Å) | 49.43 – 3.1 |
| No. of unique reflections | 10331 |
| R work (%) | 23.21 |
| R free (%) | 28.00 |
| **No. atoms** | |
| Protein | 1957 |
| Non H atoms | 2012 |
| Solvent | 55 |
| **RMS deviations from ideal stereochemistry** | |
| Bond lengths (Å) | 0.003 |
| Bond angles (°) | 0.465 |
| **Mean B factor (Å^2^)** | |
| Protein | 101.7 |
| Solvent | 102.9 |
| **Ramachandran plot:** | |
| Favoured region (%) | 92.8 |
| Allowed regions (%) | 5.9 |
| Outlier regions (%) | 1.3 |

**Table S2**. Primer sequences used for RT-qPCR experiments.

| **Gene** | **Forward** | **Reverse** |
| --- | --- | --- |
| *β-Actin* | 5’-TGACAGGATGCAGAAGGAGA-3’ | 5’-GCTGGAAGGTGGACAGTGAG-3’ |
| *Col2a1* | 5’-AGGGCAACAGCAGGTTCACATAC-3’ | 5’-TGTCCACACCAAATTCCTGTTCA-3’ |
| *Col10a1* | 5’-CATGCCTGATGGCTTCATAAA-3’ | 5’-AAGCAGACACGGGCATACCT-3’ |

**Figure S1**. **SEC-MALS analysis of the BMP-2:Noggin complex** (protein concentration of *ca.* 0.5 mg·mL^-1^). Light scattering is shown as a continuous line, whereas the molecular mass distribution (ca. 64 kDa) across the peak is shown by black dots below the peak maximum. M_w_ and M_n_ stand for the weight average and number average molecular masses, respectively, whereas the ratio Mw/Mn is known as the polydispersity index.


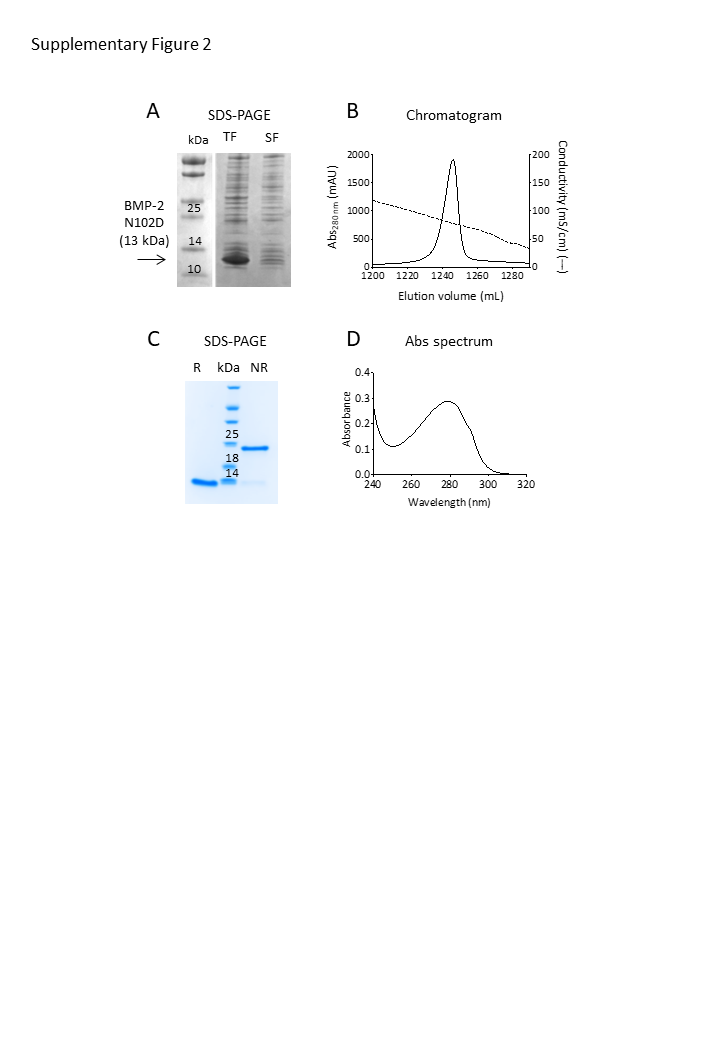


**Figure S2**. **Production of recombinant BMP-2 N102D mutant**. (A, C) Proteins were analysed following separation on 4-20% SDS-PAGE and staining with Coomassie blue. Lanes 1 to 3 in (A) correspond to the molecular weight marker, and the total (TF) and soluble (SF) fractions from induced cells, respectively. Lane 2 in (C) correspond to the molecular weight marker, whereas lanes 1 and 3 are for the purified, refolded protein, under reducing and non-reducing conditions, respectively. BMP-2 N102D was purified on a Source 15 ISO (isopropyl) column and the chromatogram is shown in (B), with mAU standing for milli absorbance units. (D) UV absorbance spectroscopy of BMP-2 N102D mutant.
